# Consistent and regular daily wearing improve bracing results: a case-control study

**DOI:** 10.1186/s13013-018-0164-0

**Published:** 2018-07-28

**Authors:** Sabrina Donzelli, Fabio Zaina, Salvatore Minnella, Monia Lusini, Stefano Negrini

**Affiliations:** 1 ISICO (Italian Scientific Spine Institute), Via Roberto Bellarmino 13/1, 20141 Milan, Italy; 2 IRCCS Fondazione Don Gnocchi, Milan, Italy; 3 Clinical and Experimental Sciences Department, University of Brescia, Brescia, Italy

## Abstract

**Background:**

In respect to the prescribed regimen and the regular daily pattern, investigate how short-term results are affected by wear time adherence in terms of hours per day.

**Methods:**

This is a case-control study. The setting is outpatient clinic. There were 168 subjects, all of whom met the inclusion criteria: adolescent idiopathic scoliosis and Sforzesco brace prescription of 18 to 23 h/day. The minimum period of follow-up was 4 months, and the maximum was 6 months, which is the average time passing between the Thermobrace (TB) adoption and out-of-brace X-ray before treatment. The brace wear adherence rate, calculated from the ratio of brace wear time with the prescription, was considered in combination with the daily pattern compliance, classified as consistent (104 patients) or inconsistent according to the abnormal distribution of Thermobrace data. The short-term results were finally explored.

**Results:**

Consistent brace wear is associated with a higher probability of improvement in curve magnitude (OR 1.96 CI 95% 1.22–3.14 chi-square 7.78 *p* = 0.0053). Inconsistent brace wear is more likely to progress (OR 0.14 CI 95% 0.30–0.75 chi-square 10.13 *p* = 0.0015). Results from the logistic regression show that the most influencing factor for improvement is Cobb degrees at the start.

**Conclusions:**

In clinical everyday activity, patients must be encouraged to consistently follow their brace wear prescription, because this attitude is clearly associated with a higher probability of improvement.

## Background

Adherence is defined as the degree to which a patient acts in accordance with the prescription of a health care provider. As attested by the results of clinical research [1], it has unquestionable implications on the effect of a therapy. The harder the treatment, the higher the risk of non-compliant patients. Brace wear for scoliosis is a very hard and complex therapy; indeed, wearing plastic for a long time and during one of the most critical phases of life, namely adolescence [2, 3], can be a real challenge.

In everyday practice, adherence is often assessed through self-completion questionnaires, or is self-reported, despite the recognized low reliability thereof [4, 5]. Therefore, objective compliance monitors were introduced, at first only for research purposes [4, 6–11]. Several studies demonstrated the reliability of temperature sensors, and, as a consequence, investigation into the effects of adherence on results started [6–11]. A prospective study on 495 scoliosis patients treated with a Boston brace concluded that the risk of curve progression and the need for surgery decreases in patients with good compliance [5]. In the same study, brace efficacy was confirmed by a multicenter RCT [12] and compliance was assessed through the use of objective monitors. A relation between dosage and results was found, thus confirming previous research: the more the brace is worn, the better the results [9–12]. Most of the results obtained with the compliance monitor showed a poor adherence to bracing [4, 10, 11, 13], as might be expected, considering the huge engagement required for such a hard therapy. Conversely, one paper subverted previous findings and demonstrated that it is possible to obtain higher adherence, even with full-time prescription. In addition, this device proved very helpful to everyday clinical activity, offering valuable help in therapeutic choices without undermining the relationship with patients and family [14]. The standardized everyday clinical usage of this temperature sensor, called Thermobrace, improves the quality of treatment through the optimization of the dosage. In fact, the use of Thermobrace in all subjects with a brace prescription has been shown to also contribute to the cognitive behavioral approach [15], which is able to enhance compliance. In a recently published survey, scoliosis patients and their families showed a very positive attitude towards these electronic devices [16].

Thinking about the biomechanics of the spine, a good consistency in terms of the duration of pauses, namely the same number of hours each day, enhances the spine’s support ability, thus improving results. Conversely, a higher variability in break lengths would undermine the support of the spine, and favors the mobilization of the spine. Actually, when the brace is on, the spine is straightened and corrected [17–19]; out of the brace, the spine collapses [20, 21] in a very short time. Based on this theory, brace prescriptions used to recommend that the brace be worn at all times, and always for the same number of hours, discouraged patients from wearing the brace less than 1 day and wearing it for an increased duration the next. Therefore, we can define the treatment as the summation of the hours of brace wear per day (with a regular daily pattern). The standard use of the TB since 2010 has demonstrated that, among patients with the same mean compliance, there are some who produced very homogeneous brace wear data and some who had days of higher break duration and days with no breaks. This can result in skewed data, with a high variability. Therefore, beyond the classification of patients based on the rate of hours per day of brace wear in relation to the prescription, a further categorization can be applied to distinguish the consistency of brace wear, thus referring to a “daily pattern compliance.”

The main hypothesis we aimed to test in this study was the following: can the hours per day of brace wear, as very close to that specified in the prescription, together with the consistency of brace wear, which means a very regular pattern of compliance, positively affect short-term results?

## Methods

### Design

This is a case-control study.

### Setting

An outpatient tertiary referral clinic, specializing in the conservative treatment of spinal deformities.

### Participants

**Fig. 1:**
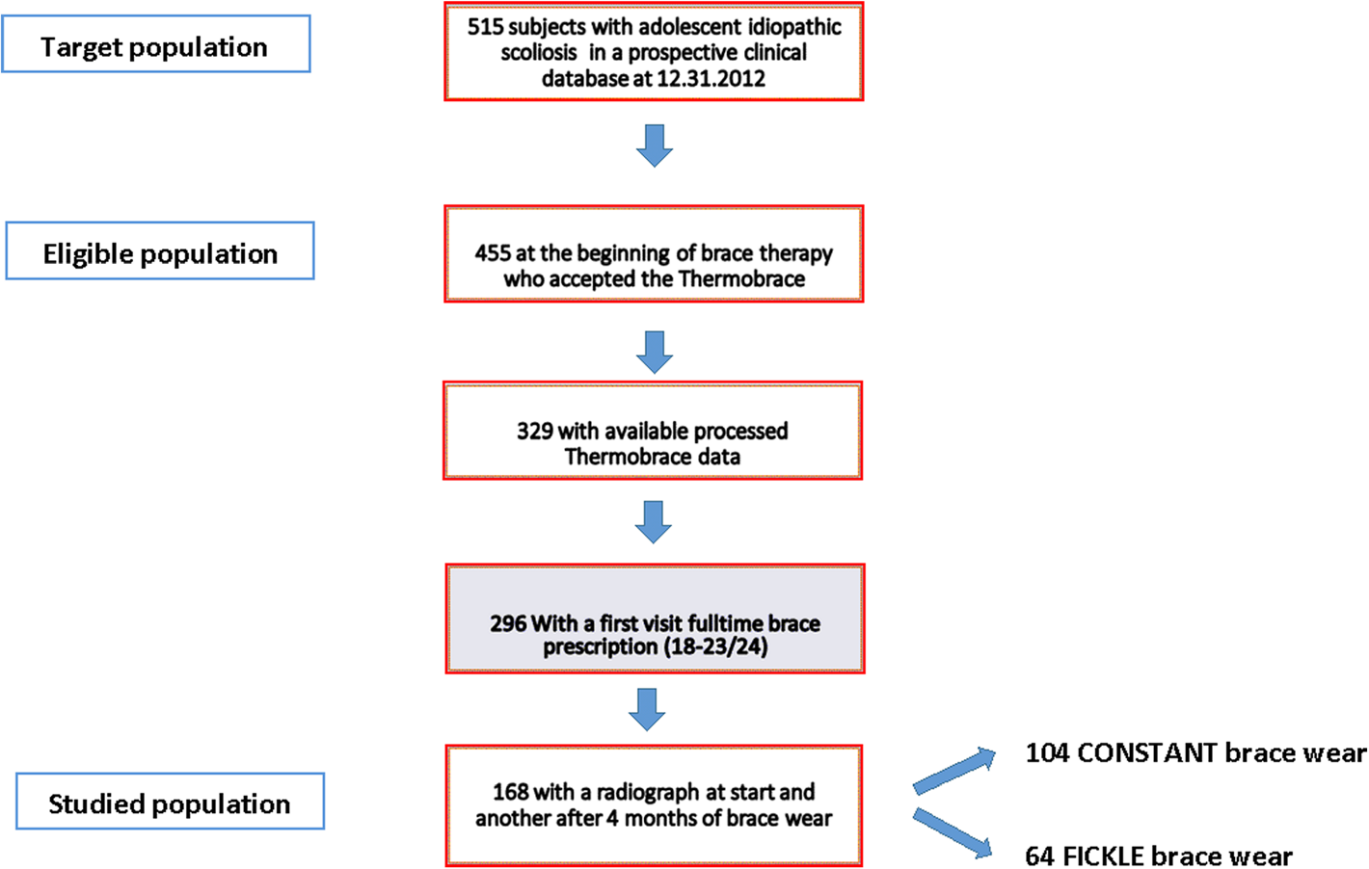
Flowchart of the selection process

The accessible population came from a prospective clinical data collection of 515 patients with adolescent idiopathic scoliosis. From January 2010 to 31 December 2012, 455 braced patients received a prescription of a Thermobrace sensor. Of these, 329 patients were provided with available Thermobrace data, consisting of one download after a minimum monitoring period of 4 months. The number of patients with a full-time Sforzesco brace prescription (from 18 to 23 h per day) was 296. One hundred sixty-eight patients had received their first, Sforzesco brace, full-time prescription and adhered to all the following inclusion criteria: idiopathic scoliosis treated with a full-time Sforzesco brace; first prescription (between 18 and 23 h per day); Thermobrace, with a voluntary and informed adoption from the beginning of the treatment; the presence of two X-rays out of the brace, one before the beginning of the treatment and one after the start of brace therapy (4 to 6 months of brace wear). Figure 1 shows the selection process.

All the patients signed their informed consent for the use of their clinical data for research purposes. All the participants voluntarily bought the Thermobrace sensor and applied this device to their brace to monitor brace wear. The local ethical committee approved the present retrospective analysis.

### Evaluations

#### X-ray monitoring

The protocol used for all adolescent idiopathic scoliosis (AIS) patients treated with braces provides for a preliminary X-ray, taken no more than 3 months before the start of the treatment. After 30 days of full-time brace wear, the “in-brace” correction is checked. After a period ranging from 4 to 6 months of brace wear, all patients undergo a radiological exam at the end of the “out of brace” period. In agreement with the SRS criteria [22], also established by the SOSORT guidelines [23], we defined the short-term results as “progressed,” “stabilized,” or “improved” if they had a change in the curve of at least 5° Cobb.

#### Thermobrace monitor

The Thermobrace is a heat sensor which measures brace temperature and includes specially developed software designed to define if the brace is being worn or not, using a specific algorithm [8, 10, 13, 14]. At each visit, thanks to the data acquired by the Thermobrace, which was processed via specifically developed software, it was possible to check both parts of the treatment: “brace wear adherence,” representing the hours of brace wear per day in relation to the hours prescribed (rate value), and “daily pattern compliance,” which has been defined by the consistency index.

### Treatment

All the participants were treated with a Sforzesco brace, a rigid polycarbonate orthosis in two pieces, held together by a vertical aluminum bar, developed according to the SPoRT concept [18]. A number of previously published papers have demonstrated the efficacy of this brace, even at the end of growth and in very severe curves [24] and its superiority to the Lyon brace [25, 26].

#### Brace therapy management

At the end of the first visit, and during the brace check at the orthotics’ facility, the involved professionals explained to the patient and the family the usefulness of the Thermobrace [14]. The patients and their family decided autonomously whether or not to buy this electronic monitor. At each exercise session, the physiotherapist checks the use of the brace, discussing and solving any problems or troubles experienced by the patient and the family. During the clinical follow-up visit, the doctor may also check the use of the brace, to discuss and try to solve the major troubles related to brace wear and to adjust the brace dosage prescription according to the real use of the brace.

### Groups

#### Categorization process

As already in previous research [14], participants were classified according to “brace wear adherence.” Regarding the prescribed regimen, “brace wear adherence” percentages were calculated from the ratio of brace wear time measured by the Thermobrace (the hours of brace wear in relation to the hours prescribed). Therefore, if the calculated percentage of adherence was above 90%, between 70 and 89% or below 70%, patients were classified as high, medium, or low compliers, respectively. These three categories were defined according to the first and third quartile of the distribution of the values found in the two groups considered: consistent and inconsistent brace wear. In the consistent daily pattern group, the first quartile was 88.7% and the third quartile was 95.5% while in the inconsistent daily pattern brace wear group they were, respectively, 67.3 and 93.5%. This distribution highlights the high level of compliance, which characterize the Isico setting, as confirmed by previous results from the Thermobrace [14].

**Fig. 2:**
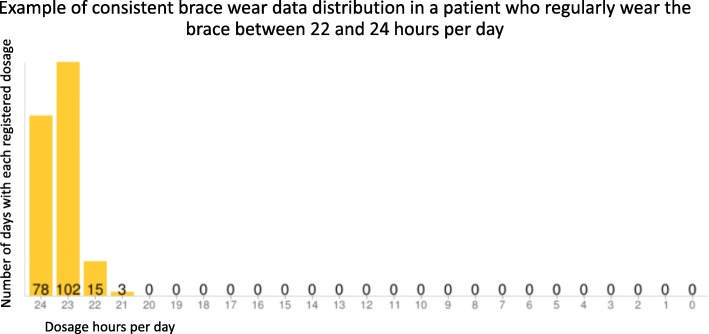
An example of consistent brace wear Thermobrace output

**Fig. 3:**
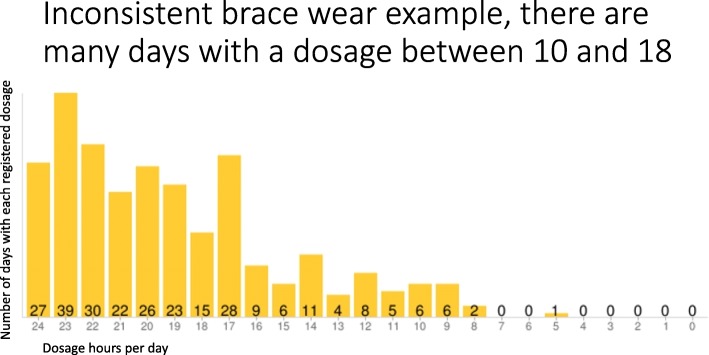
An example of inconsistent brace wear Thermobrace output

Figure 2 offers an example of consistent brace wear Thermobrace output, while in Fig. 3 there is an example of inconsistent brace wear. Specifically developed software for processing Thermobrace data allows us to check both parts of the treatment: in percentages, we may define “brace wear adherence” as a function of both hours/day adherence, in respect to the hours prescribed, as well as “daily pattern compliance,” which represents the consistency of brace wear. The short-term results were finally explored by considering brace wear adherence combined with the daily pattern compliance. Therefore, these electronic devices can provide not only the rate of brace wear in respect to the prescribed time, but also a huge amount of data accessible to all the professionals involved in the treating team, and maybe in the future also the parents and family.

To identify subjects with a greater scattering of data and to distinguish them from patients with more homogeneous data, measures of variability were applied. The abnormal distribution of data, associated with skewness, and the presence of outliers determined the choice of the interquartile range as the best and more feasible measure of variability. The interquartile range is the distance between the first and the third quartile and represents the boundaries of the middle 50% of the distribution. The average brace wear per month, as registered by the Thermobrace, was considered, and the consistency index was defined by the difference of at least 1 h in the interquartile range (the applied formula to define the consistency index is the following: third quartile median minus the first quartile median > 1). This formula was used to define patients as either “inconsistent” or “consistent” in their brace wear, with respect to daily pattern compliance.

#### Data analysis

The Cobb degrees measured on the first X-ray, conducted out of the brace, after a brace wear period ranging from 4 to 6 months, were compared to the curve magnitude at the beginning of the therapy. These results were categorized according to the 5° Cobb threshold, agreed by scoliosis experts and recommended by the SRS, to define the final outcome as improved, stabilized, or progressed [22, 27, 28]. Daily pattern compliance (defined as consistent or inconsistent brace wear) was considered a significant influencing factor, together with brace wear adherence, when measuring the risk of worsening. In the present study, we examined two correlated samples. Subjects belonging to the same sample were used as their own controls. The basic assumption of independence required by a chi-square test could not be respected, and McNemar’s test was deemed the best measure of association for analyzing categorical variables by using a 2 × 2 table that involves correlated samples. Therefore, McNemar’s chi-square test, the odds ratio of progression, and stabilization with a 95% confidence interval (IC95) were used. The brace wear adherence (defined as high, medium, and low) was evaluated according to the daily pattern compliance (consistent or inconsistent) to find the risk of progression or stabilization (which can be considered the worst outcome). The probability of improvement (the best possible outcome) was also investigated. In a secondary analysis, for the risk of progression, a multivariate logistic regression with adjustment for factors significantly associated with progression risks was applied. The confounders considered were gender, Risser score, and Cobb degrees at the start. These were analyzed independently and combined.

## Results

Out of the 168 patients (105 females), 104 were classified according to the daily pattern compliance as having consistent brace wear, and 64 as having inconsistent brace wear. We did not find statistical differences in all primary parameters between consistent and inconsistent brace wearers, except for the gender distribution, which showed a higher rate of males in the fickle brace wear group (Table 1). Table 2 shows the results for the two groups of different daily pattern compliance, according to brace wear adherence (defined as high, medium, or low).

**Table 1:** Primary parameters in the two groups considered

	Inconsistent brace wear	Consistent brace wear	*p* values
Mean age (SD)	13.7 (1.8)	12.9 (1.9)	NS
Mean Cobb degrees (SD)	41.1 (11.8)	41.7 (11.2)	NS
Males	50 (78.1%)	13 (12.5%)	< 0.001
Females	14 (21.9%)	91 (87.5%)	< 0.001

**Table 2:** Results of the two groups of different daily pattern compliance, according to the brace wear adherence (defined as high, medium, or low)

	Improved *n* (%)			Stabilized *n* (%)			Progressed *n* (%)		
Consistent daily pattern *n* (%) 104 (61.9%)	55 (52.8%)			38 (36.6%)			11 (10.6%)		
	Hours/day compliance*			Hours/day compliance			Hours/day compliance		
	High	Medium	Low	High	Medium	Low	High	Medium	Low
	29 (54.1%)	26 (47.3%)	0 (0%)	13 (35.2%)	25 (65.8%)	0 (0%)	1 (9.6%)	10 (90.4%)	0 (0%)
Inconsistent daily pattern *n* (%) 64 (38.1%)	24 (39.1%)			36 (54.7%)			4 (6.3%)		
	Compliance			Compliance			Compliance		
	High	Medium	Low	High	Medium	Low	High	Medium	Low
	7 (10.9%)	12 (18.8%)	5 (9.4%)	7 (10.9%)	16 (23.4%)	13 (20.3%)	0 (0%)	3 (4.7%)	1 (1.6%)

* Percentage of adherence was above 90%, between 70 and 89% or below 70%, patients were classified as high, medium, or low compliers, respectively

Subjects with an adherence of below 94% (classified as low and medium compliers), and with inconsistent daily pattern compliance, are more likely to progress than those with a consistent daily pattern compliance (OR 0.19 CI 95% 0.096–0.37 chi-square 23.12 *p* = 0.0000).

The results concerning the measures of association showed that consistent daily pattern compliance is associated with a higher probability of improvement in curve magnitude after the first few months of treatment (OR 1.96 CI 95% 1.22–3.14 chi-square 7.78 *p* = 0.0053). Consequently, inconsistent daily pattern compliance is more likely to progress (OR 0.14 CI 95% 0.30–0.75 chi-square 10.13 *p* = 0.0015).

Adherence was very high in the consistent brace wear group. 81.70% of patients were high compliers, which means over 95% of the prescription. In the inconsistent daily pattern compliance group, the proportion of subjects with high adherence was 21.80% (*p* < 0.0001); among these, 50% improved and 50% remained stable.

Inconsistent daily pattern compliers tended to be categorized as medium or low compliant. Consistent daily pattern compliers were generally adherent to the prescription, regardless of the number of hours prescribed. Inconsistent daily pattern brace wearers were characterized by a negative correlation between the hours of prescription and compliance. Males were more likely to be inconsistent compliers than females. Indeed, males were more frequent in the inconsistent daily pattern compliance group (78.13% versus 12.50%; *p* < 0.05). The comparison of adherence according to curve magnitude demonstrated that the severity of the curve did not affect the adherence level or quality. Consistency in brace wear showed itself to be linked to a higher brace wear compliance, which is of relevance when determining short-term results together with the amount of compliance.

Adjusted for the brace wear adherence and the daily pattern compliance, the results of the logistic regression showed that the most influential factor for improvement was the Cobb degrees at start. Subjects with less than 35° at the beginning of the therapy were almost four times more likely to obtain a significant regression of curves (*p* = 0.001). Risser and gender had no significant influence on the results.

## Discussion

Despite the hard engagement requested during brace therapy to ensure good results, our present and previous results confirm that it is possible to obtain a higher adherence to brace wear [14] and that teamwork is fundamental to obtaining such adherence to treatment [29]. When referring to brace therapy, dose and goals are strictly related. In scoliosis treatment, the main goal of brace therapy can change according to the curve magnitude and other key factors, all of which influence curve progression. Therefore, in some cases, an improvement is needed, while in other cases, a stabilization of the curves is enough [23]. According to the scientific literature, the main outcome to be considered in the analysis of brace wear results is progression avoidance. The present research has deepened the issue of the best dosage and management of brace wear for AIS patients, by considering as main outcome not only the stabilization of curves, but also the possible improvement of curves [27].

The standardized application of compliance monitors has already demonstrated various advantages [14, 30]. Very recently, the relationship between adherence to the prescribed regimen and the results of the therapy was confirmed; but, more interestingly, Reinker and colleagues found that the awareness of being monitored and the possibility of discussing the objectively measured wearing time with the treating team improve compliance [31].

The present study is the first in which the standardized application of the Thermobrace is used to analyze the short-term effects of brace therapy in respect not only to brace wear adherence, but also to daily pattern compliance. According to the present research, “consistent brace wear” plays a significant role in determining satisfactory results, compared to high adherence alone. Hence, high-quality brace wear is defined by both high adherence to the brace wear prescription and consistency in the daily pattern compliance. Very often, on any particular day, adolescents with scoliosis prefer to wear the brace for fewer hours than prescribed, and to recover time in the brace the day after. Parents often also prefer this kind of behavior. It involves 1 day of “maximum sacrifice” in order to gain some more hours of freedom. This behavior appears to favor brace adherence but, as demonstrated above, it can also affect results. In light of the present results, asking for slightly fewer hours of brace wear, but with the compromise of a high level of consistency, would be a better approach.

The Sforzesco brace has demonstrated its efficacy in curve improvement in previously published observational studies [18, 24, 32, 33]. It is possible that brace efficacy confounds the present results. In fact, a more significant difference is seen in the comparison of improvement in respect to stabilization and worsening, so that the entire sample considered is unbalance in the improvement direction, and the rate of progress was very low. This is the reason why the measure of association produced OR values below one. All the statements below justify the choice of the main outcome of the actual research: the rate of improvement being associated with a high adherence to the brace wear prescription. The logistic regression analysis showed that the major confounder of the current results is the Cobb degrees at start; as expected, the milder curves are more likely to regress, while more severe curves tend to stabilize, rather than improve.

One of the hypotheses, which is relevant to the evidence presented, addresses the in-brace correction and the postural collapse already documented when out of the brace [34]. At the start of breaks, the spine collapses quickly, thus losing its correction. Therefore, the longer the break, the higher the correction lost. At the end of this phase, the brace will, again, correct the spine. As the number of breaks increases, the mobilization of the spine can increase accordingly. This theory is the basis of the traditional recommendation that patients wear the brace regularly every day, respect their prescription very closely, and engage in the same number of hours of brace wear. Prior research in the field of postural and clinical changes at brace weaning [20, 21] have shown that postural collapse is correlated to the length of brace weaning and to the flexibility of the spine.

One of the main limitations of the present study was the inclusion of AIS patients, regardless of curve magnitude, and the inclusion of very severe curves, together with milder scoliosis. A heterogeneity of data can produce biased results, and caution is needed in the interpretation of results thereof. During study planning, we decided to avoid any curve magnitude selection criteria, to guarantee a larger sample for the purpose of focusing on the main concerns: the effect of brace wear adherence combined with daily pattern compliance on short-term results. This choice was supported by previous findings in which brace wear was not influenced by curve magnitude nor curve type [14, 35]. In addition, we strongly agreed that this behavior would have enhanced the generalizability of the results. Curve type was not considered among the influencing variables, because it showed an unbalanced distribution in the sample. The lateral view of the spine was not available for all the included subjects; therefore, global sagittal balance parameters were not considered, together with curve magnitude.

## Conclusion

In clinical everyday activity, patients must be encouraged to be consistent and to follow their prescription for brace wear closely, because this attitude is clearly associated with a higher probability of improvement. Even with less adherent brace wear, consistency can be rewarded with better short-term results. During brace treatment, all the members of the team must endorse consistent and rigorous brace wear, as it has been clearly demonstrated that this attitude will produce better results.

The actual findings offer some contribution to clinical decision-making. Further analysis using long-term monitoring will consider all the possible effects associated with adherence to the treatment and the final results. The usefulness of the everyday standardized application of the Thermobrace has been confirmed once again.

## Abbreviations

AIS: Adolescent idiopathic scoliosis
RCT: Randomized controlled trial
SOSORT: International Society on Scoliosis Orthopaedic and Rehabilitation Treatment
SPoRT: Symmetric, patient-oriented, rigid, three-dimensional, active
SRS: Scoliosis Research Society
